# Mutation at the entrance of the quinone cavity severely disrupts quinone binding in respiratory complex I

**DOI:** 10.1038/s41598-023-47314-2

**Published:** 2023-11-21

**Authors:** Jason Tae Yi, Panyue Wang, Alexei A. Stuchebrukhov

**Affiliations:** Department of Chemistry, University of California at Davis, One Shields Avenue, Davis, CA 95616 USA

**Keywords:** Biophysics, Cell biology, Computational biology and bioinformatics, Diseases

## Abstract

In all resolved structures of complex I, there exists a tunnel-like Q-chamber for ubiquinone binding and reduction. The entrance to the Q-chamber in ND1 subunit forms a narrow bottleneck, which is rather tight and requires thermal conformational changes for ubiquinone to get in and out of the binding chamber. The substitution of alanine with threonine at the bottleneck (AlaThr MUT), associated with 3460/ND1 mtDNA mutation in human complex I, is implicated in Leber's Hereditary Optic Neuropathy (LHON). Here, we show the AlaThr MUT further narrows the Q-chamber entrance cross-section area by almost 30%, increasing the activation free energy barrier of quinone passage by approximately 5 kJ mol^−1^. This severely disrupts quinone binding and reduction as quinone passage through the bottleneck is slowed down almost tenfold. Our estimate of the increase in free energy barrier is entirely due to the bottleneck narrowing, leading to a reduction of the transition state entropy between WT and MUT, and thus more difficult quinone passage. Additionally, we investigate details of possible water exchange between the Q-chamber and membrane. We find water exchange is dynamic in WT but may be severely slowed in MUT. We propose that LHON symptoms caused by 3460/ND1 mtDNA mutation are due to slowed quinone binding. This leads to an increased production of reactive oxidative species due to upstream electron backup at the FMN site of complex I, thus resulting in a mt bioenergetic defect.

## Introduction

### Respiratory complex I and the narrow bottleneck at the entrance to the Q-chamber

NADH:ubiquinone oxidoreductase, or complex I, is the first and largest respiratory enzyme within the electron transport chain, which plays a major role in cellular bioenergetics^1,2^. It couples the oxidation of NADH and the reduction of ubiquinone to proton translocation, which contributes to the creation of proton gradient that drives ATP synthesis. Recent structural studies^3–13^ of the enzyme have revealed molecular details that suggest possible molecular mechanisms of its redox-driven proton pumping^14–16^. Several models have been considered in the literature (see, refs ^11,14–20^), but there is no consensus yet on the complete molecular mechanism of the enzyme. Ubiquinone binding and reduction is key for the mechanism of complex I.

**Figure 1:**
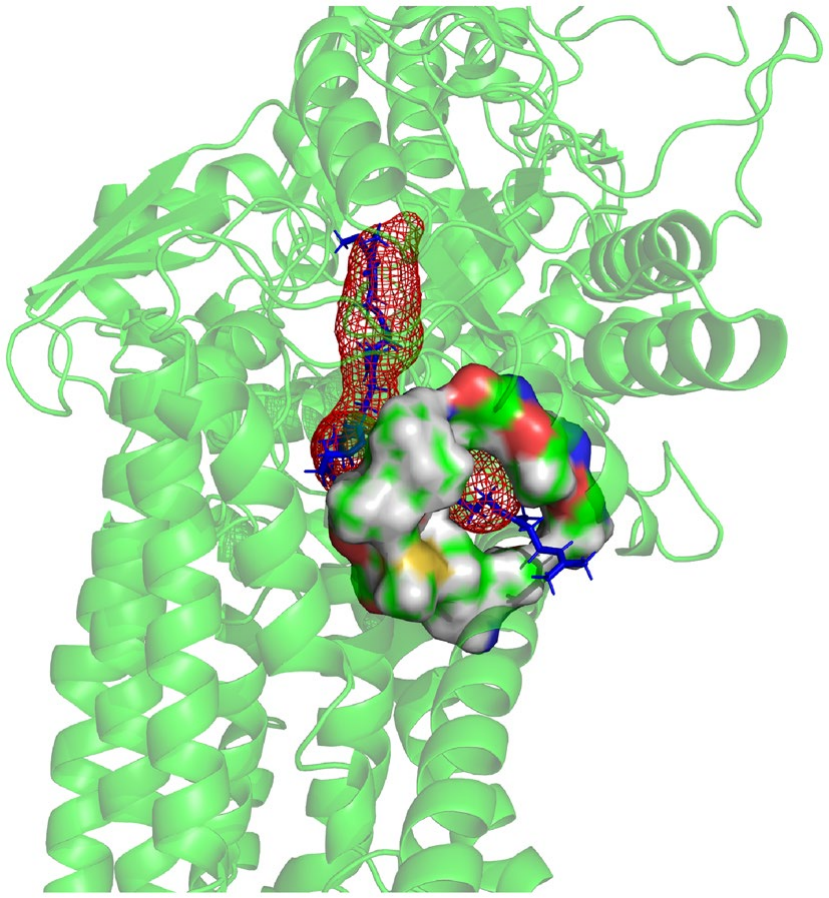
Visualization of the Q-chamber and narrow bottleneck in complex I, with ubiquinone bound to complex I. Quinone is shown in blue, and the donut-shaped bottleneck is shown with surface rendering using PyMOL^23^. The Q-chamber was calculated using the CAVER^24^ add-on.

In all organisms, the structure of the core part of the enzyme reveals an almost 30 angstrom tunnel-like void for ubiquinone binding (Q-chamber) that leads from the N-edge of the membrane up to the N2 FeS cluster^21^. The Q-chamber is comprised of subunits Nqo4/NDUFS2, Nqo6/NDUFS7, and Nqo8/ND1 (*T.thermophilus*/Human notation). It is believed quinone enters the cavity, migrates to the N2 cluster, and after reduction exits the cavity as a quinol^1^. The structure of the enzyme shows that the entrance to the Q-chamber forms a narrow bottleneck which is conserved in all organisms^21,22^. The entrance to the Q-chamber is formed by a specific crossing of TM1, AH1 and TM6 helixes in the ND1 subunit. Previous work has shown the bottleneck presents a high energetic barrier for quinone’s passage of the bottleneck, indicating conformational changes are required to allow quinone to bind to complex I^21^. A squeeze-in mechanism has been proposed, where dynamic thermal conformational fluctuations allow quinone to get in and out^22^ (Fig. 1)

In all resolved structures, the bottleneck is too narrow to allow free quinone passage. The reason for the bottleneck to be evolutionary maintained, and to impede a free binding path to catalytic chamber, in striking contrast to other ubiquinone binding sites of the respiratory chain, remains obscure. As a hypothesis, the quinone shuttle model has been proposed, where one quinone molecule stays bound in the catalytic cavity without the need to pass the narrow bottleneck^20^ however, there is no yet direct experimental evidence for such a mechanism and the path for electron transfer down the chain remains unclear. Here we assume the conventional model in which quinone is getting in and out of the catalytic cavity and further investigate the mechanism of bottleneck passage.

Previous studies have also discussed the role of water in quinone binding; it can either stabilize ionic networks in the Q-chamber or to reduce hydrophilic-hydrophobic interactions through water expulsion^25,26^. Sazanov et. al. have suggested a “domino effect” mechanism for complex I, where water exchange between the Q-chamber and membrane assists quinone to enter and exit its binding site (see ref. ^26^). Individual water molecules have been detected experimentally inside the Q-chamber of *Y. Lipolytica* complex I^13,20^, though there is yet uncertainty in the extent of overall hydration in the Q-chamber. The bottleneck's tight geometry may affect water exchange upon quinone binding and thereby control overall quinone binding kinetics. Here we address some of the kinetic issues of water exchange between the Q-chamber and the membrane.

### Leber’s hereditary optic neuropathy

Leber’s Hereditary Optic Neuropathy (LHON) is a maternally inherited disease characterized by severe visual loss^27–30^. The disease is commonly observed in males in their second or third decade of life. Complex I is implicated in the pathogenesis of LHON. Symptoms are believed to arise due to a combination of bioenergetic defect and increased production of reactive oxidative species (ROS)^28–31^. Several mutations have been observed in LHON patients, but three mutations in complex I account for almost 95% of all LHON cases. They are mitochondrial DNA (mtDNA) 3460/ND1, 11778/ND4, and 14484/ND6^29^.

**Figure 2:**
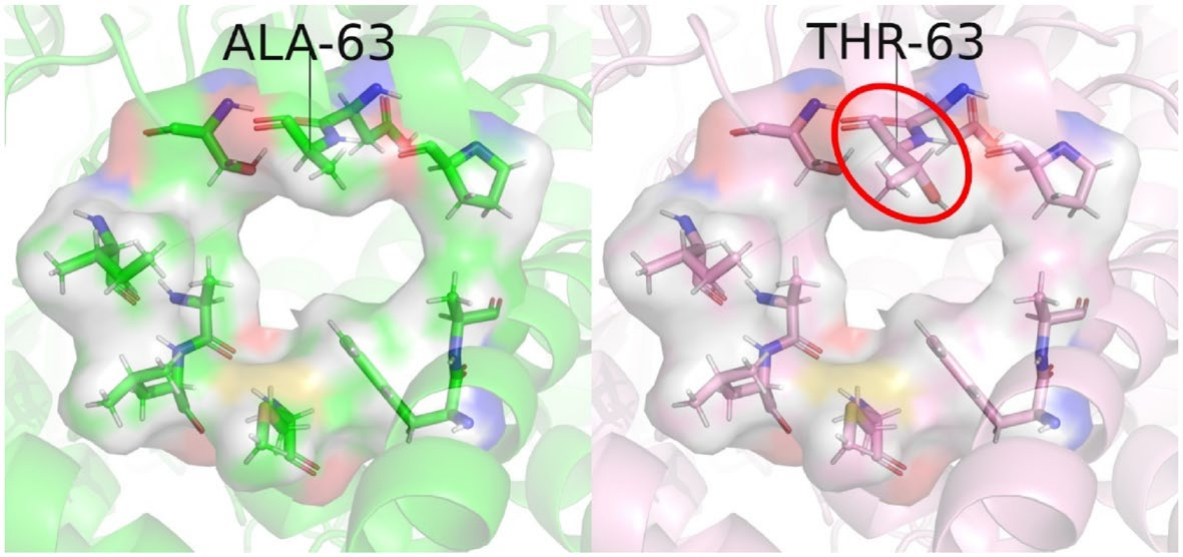
Narrow bottleneck of wild type *T. Thermophilus* (PDB: 4HEA) (left), and of the AlaThr MUT (right). Alanine 63 (Alanine 52 in human complex I) is substituted with threonine (see ref ^27^, Ala52Thr in ND1). *T. Thermophilus* is our model system for all calculations, as it is similar to the human complex I bottleneck structure (see SI Figs. S3,S4). Importantly, the human bottleneck is not obstructed by any accessory subunits in the enzyme, so we anticipate the binding mechanism of Q to complex I to be comparable in both species. It is visually apparent that the substitution from alanine to threonine increases obstruction of the bottleneck.

The 3460/ND1 mtDNA mutation has the highest biochemical severity^30,31^. Complex I is fully assembled and active in patients with this mutation, but it has been shown to significantly increase ROS generation in neuronally differentiated cells^32^. In human complex I, this mutation results in the substitution of alanine with threonine at position 52 in ND1 subunit^27,28^. Alanine 52 is at the Q-chamber entrance that forms the bottleneck (see Fig. 2). Its substitution with threonine is believed to disrupt electron transfer by obstructing the Q-chamber entrance. Hereafter, we will refer to the 3460/ND1 mtDNA mutation as AlaThr MUT, or simply MUT.

In the present study, we use computer simulations to investigate how the AlaThr MUT affects quinone binding to complex I. We demonstrate that quinone passage through the bottleneck is slowed almost tenfold in MUT relative to the wild type variant. We show the reduced passage rate is due to the reduced cross-section area of the bottleneck, leading to the transition state entropy reduction and corresponding higher free energy barrier of passage. Additionally, we explore the energetics of water exchange through the narrow bottleneck when quinone is bound, which indicates that water passage is significantly slowed down in MUT relative to the wild type variant. Together, our study suggests that AlaThr mutation at the bottleneck of Q-chamber results in a slowed quinone binding, leading to the reduction of FMN which gives rise to an increased production of ROS; this explains the nature of 3460/ND1 mtDNA mutation bioenergetic defect in humans.

## Results

### Cross-section area of the bottleneck

**Figure 3:**
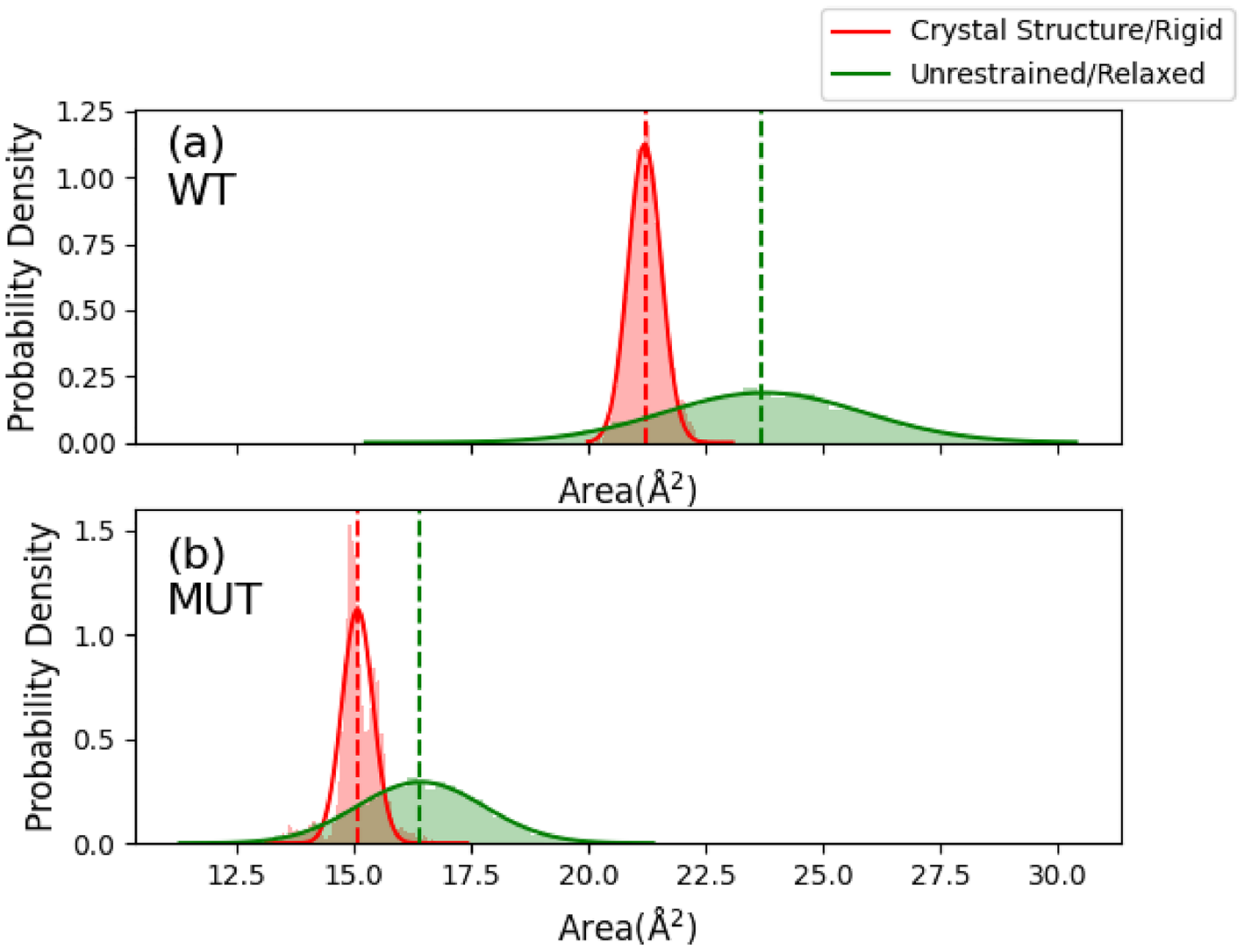
The cross-section area distributions of the *T. Thermophilus* the wild type (**a**, left) and AlaThr (**b**, right) variant bottlenecks from two MD simulations. The first simulation restrains all bottleneck atoms near their positions in the crystal structure (crystal structure/rigid, red). The second simulation has zero position restraints on the bottleneck atoms (unrestrained/relaxed, green).

We first performed structural analysis of the bottleneck (Fig. 3 and Table 1). We set up two molecular dynamics (MD) simulations of the bottleneck. In the first simulation, hereafter denoted “crystal structure/rigid," atoms were strongly restrained to keep the geometry of the bottleneck restricted to that of the PDB structure. In the second simulation, hereafter denoted "unrestrained/relaxed," no position restraints were applied to the bottleneck atoms. This was done to allow the bottleneck to relax and explore its entire MD-simulated conformational space. However, we noticed the relaxed state is somewhat different from that reported in PDB structure, and obviously represents an artifact of the force field. Normally, this does not cause much global difference, however here the minor details become important due to the tight nature of the bottleneck. Together, these two simulation conditions will allow us to capture the full range of allowed cross-section areas.

**Table 1:** Summary of bottleneck cross-section area from the distributions plotted in Fig. 3, along with the percent change between the wild type (WT) and the AlaThr (MUT) bottleneck variants.

Restraint	WT	MUT	Percent change
Crystal structure/rigid (Å^2^)	21.23 ± 0.01	15.10 ± 0.01	− 28.9%
Unrestrained/relaxed (Å^2^)	23.69 ± 0.05	17.46 ± 0.04	− 26.3%

We see the average cross-section area, A, and 95% confidence intervals of the wild type bottleneck is A_rigid_ = 21.23 ± 0.01 Å^2^, and A_relaxed_ = 23.69 ± 0.05 Å^2^. The average cross-section area and 95% confidence intervals of the AlaThr MUT are A_rigid_ = 15.10 ± 0.01 Å^2^, and A_relaxed_ = 16.38 ± 0.03 Å^2^.

### Bottleneck deformation during quinone passage

**Figure 4:**
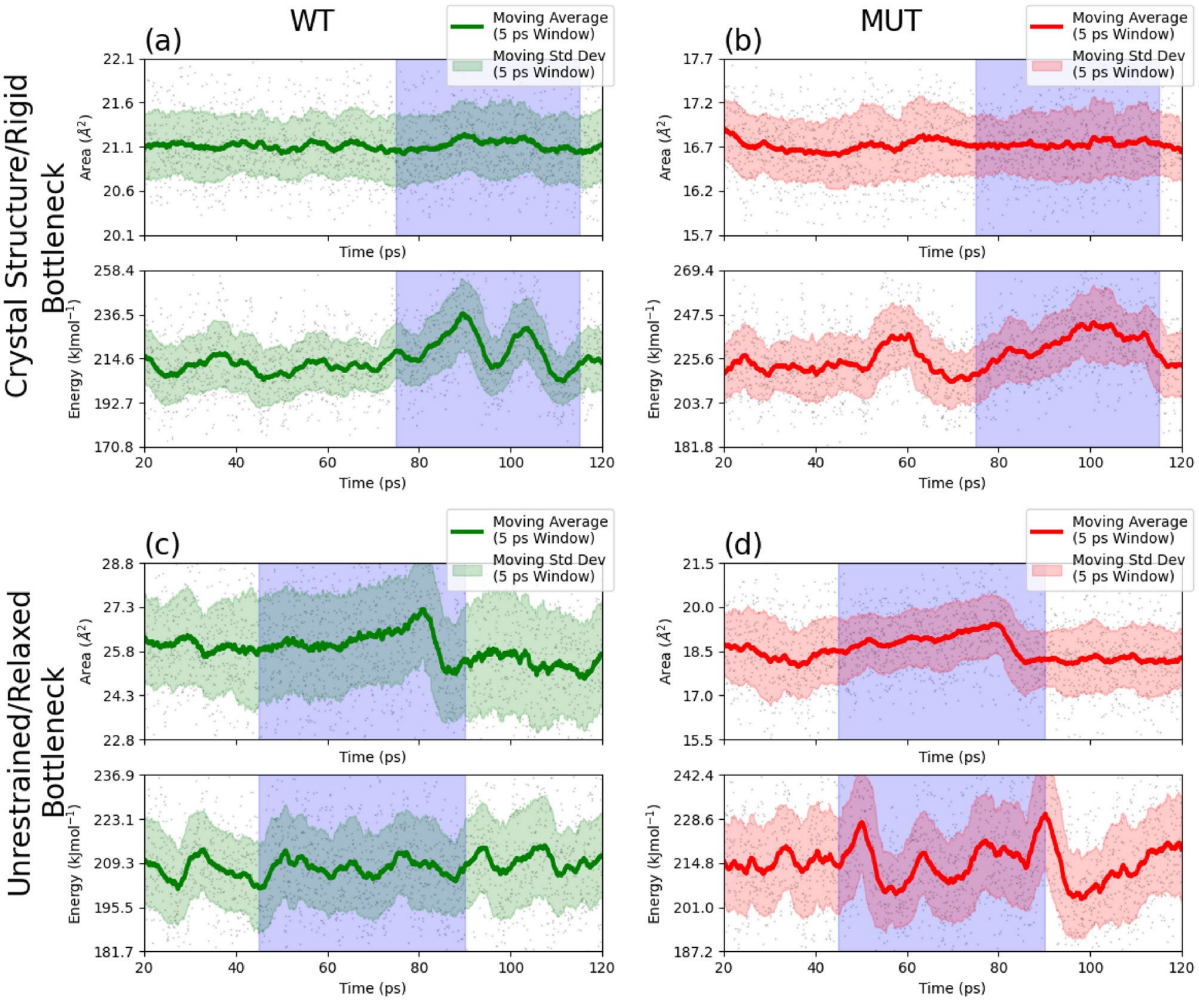
Bottleneck and quinone head group deformation energy during a series of steered MD simulations. The left column is the wild type bottleneck (green), and the right column is the AlaThr MUT bottleneck (red). The top row contains simulations performed with a rigid bottleneck, and bottom row contains simulations performed with a relaxed bottleneck. In each quadrant, the top graph is the cross-section area of the bottleneck, and the bottom graph is the bonded energy of the Q1 head group. Regions highlighted in blue roughly correspond to the time in the simulation when quinone is passing through the bottleneck. For comparability, note that the cross-section area in the top row span about 1 Å^2^, whereas the cross-section area in the bottom row span about 5 Å^2^.

To quantify the extent of deformation of the bottleneck during quinone passage, we ran several steered MD simulations. During these simulations, Q_1_ (ubiquinone head group with only one isoprenyl tail unit) was pulled at a rate of 0.04 Åps^−1^ from a position deep inside the Q-cavity of (approximately, the 2’ position, as denoted in Fig. 3 of in Ref. ^33^, see also SI Fig. S16) until dissociation. Simulations were performed in rigid and relaxed conditions for both the wild type and AlaThr MUT. During the simulation, we monitored the bottleneck cross-section area, and the bonded energy of the Q1 head group (to estimate the energetic cost of quinone deformation during the passage). The cross-section area was calculated using the algorithm described in SI section b, and the bonded energy of the head group was calculated by Gromacs energy reruns. Together, these two metrics illustrate how the geometry of the bottleneck impacts quinone passage (see additional discussion in Methods). Results are shown in Fig. 4. It is seen that in the rigid simulations, the passage of the bottleneck is associated with increased strained energy of the quinone head group. In the relaxed simulations, we see the bottleneck deforms instead, leading to increased cross-section area of the bottleneck. Together, these results demonstrate the quinone head group and bottleneck non-negligibly interact with one another. The tighter geometry of MUT leads to exacerbation in both deformations.

### Free energy barrier of quinone bottleneck passage

To quantify the obtained results shown in Fig. 4, we calculated a modified form of free energy of quinone passage through the bottleneck. To avoid the uncertainty of evaluation of deformation energy of the whole protein structure during the passage, we calculated the free energy excluding non-bonded potential of interaction of quinone and protein, thus focusing only on the more reliable geometric aspect of the problem. That is, we define the free energy of passage as follows:

1 $$\widehat{G}=G\left(\xi \right)-{U}_{\text{NB}}\left(\xi \right)$$

where ξ denotes the reaction coordinate, G(ξ) denotes the Helmholtz free energy, U_NB_(ξ) denotes the non-bonded potential between the protein and quinone head group along ξ. We are interested in understanding how the geometry (more reliable structural aspect) of the entrance bottleneck impacts quinone passage.

AlaThr MUT is a nonlethal mutation, meaning we suspect only minor differences to exist between the WT and MUT transition state quinone binding barrier. Uncertainty in the non-bonded potential of interaction, ${\sigma }_{{\text{N}}{\text{B}}}$, is proportional to $RT\surd N$, where N denotes the number of atoms. Including the non-bonded potential of interaction, and taking N = 150–200 atoms, we see ${\sigma }_{{\text{N}}{\text{B}}} \approx$ 30–35 kJ mol^−1^. Any changes in our free energy barrier which are below this uncertainty, such as minor changes resulting from the AlaThr MUT, will be lost in the noise. Therefore, we subtract the non-bonded interactions as these terms to better highlight the changes in our free energy barrier resulting from the bonded and entropic terms of free energy. In doing so, we are measuring how the geometry of the bottleneck changes the free energy of quinone passage between the two variants. We acknowledge that nonbonded interactions play a nontrivial role in defining the barrier height of the bottleneck and have quantified these contributions previously in ref ^21^; our previous studies have demonstrated large uncertainty of such energy contributions, which we aim to avoid here. Thus, our results represent a lower boundary of the effect.

**Figure 5:**
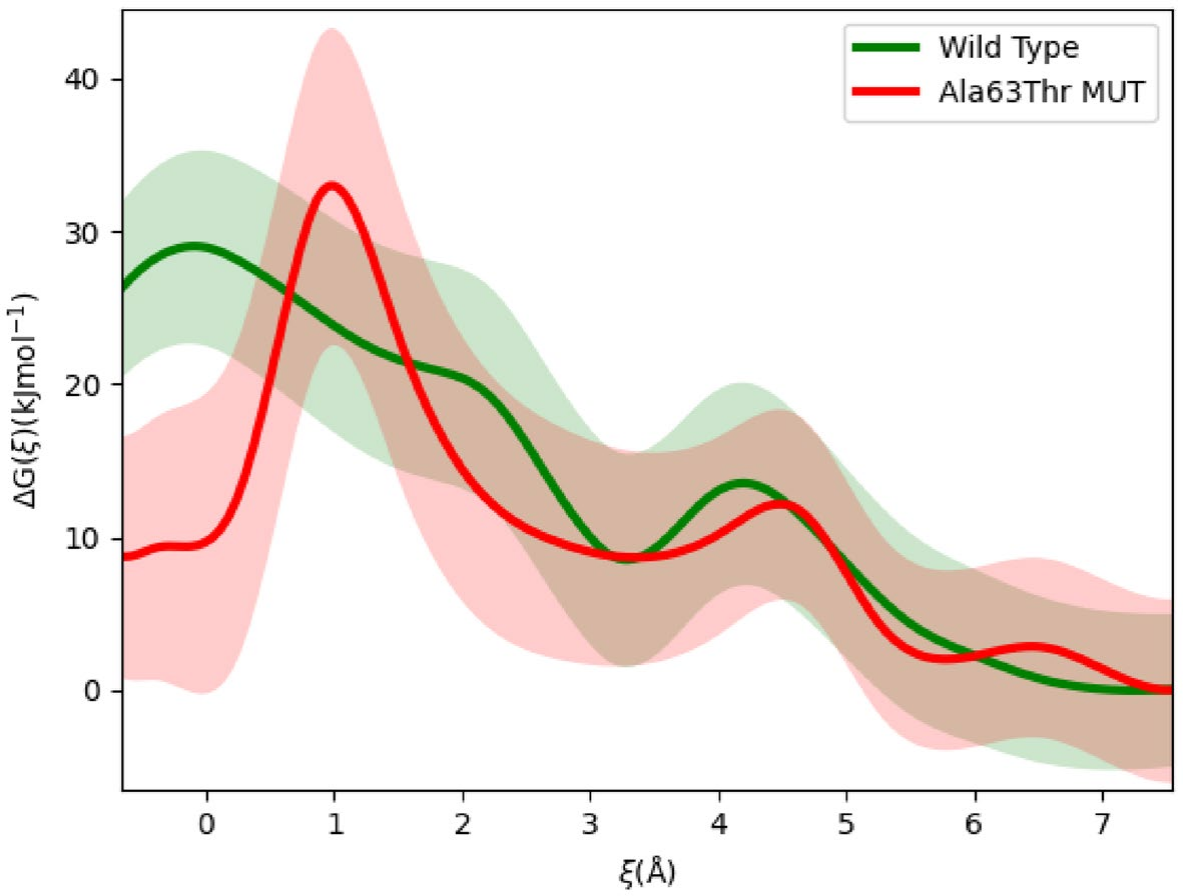
Free energy of quinone passage through the bottleneck for the wild type WT (green) and AlaThr MUT variant (red) with uncertainties.

The reaction coordinate, ξ, was chosen to be a spline representative of the path taken by the head group during our steered MD simulations. Free energy profiles were calculated for both WT and MUT, are shown in Fig. 5.

The free energy plotted in Fig. 5 is from Eq. 1. Therefore, the free energy differences result purely from the geometry of the entrance bottleneck (and its effect on the entropy) and head group deformation. We see there is a 5 to 10 kJ mol^−1^ difference in the passage barrier between the two variants.

### Water exchange through the bottleneck (when quinone is bound in Q-chamber)

The bottleneck’s tight geometry impacts quinone passage, and therefore we should expect water exchange—when the quinone head or a tail occupies the bottleneck—to be impacted as well. We speculate that water exchange may play a critical role in the binding process and thus can regulate the overall binding kinetics.

**Figure 6:**
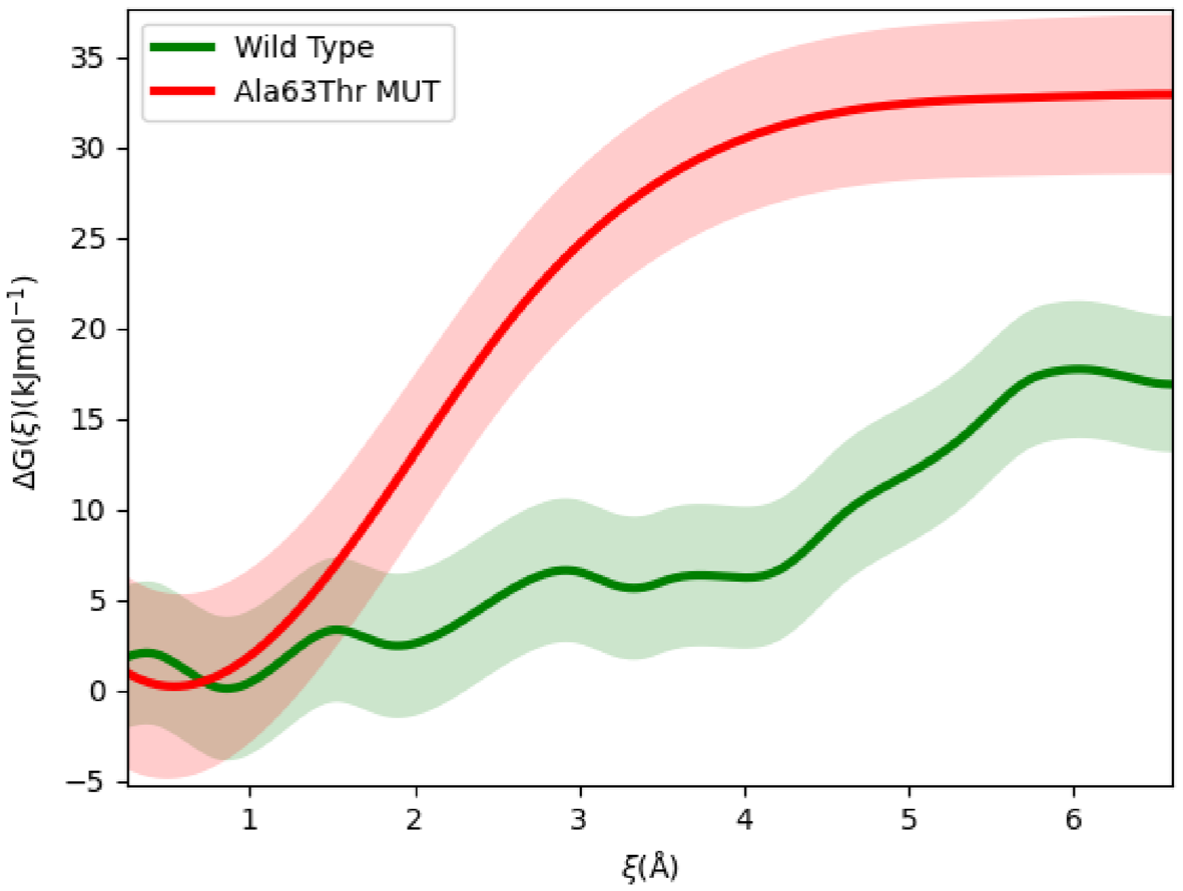
Free energy of water passage through the bottleneck when quinone is bound for the wild type (green) and AlaThr variant (red) with uncertainties. The free energy plotted here is from Eq. 1. We see there is a 15 kJ mol^−1^ difference in the dissociation barrier between the two variants.

To explore our proposal, we calculated the modified free energy of water passage through the bottleneck. Water was placed in the interior of the Q-chamber, near the bottleneck. Ubiquinone (Q_10_) in calculations was bound in its binding site, with about one isoprenyl tail unit sticking out of the bottleneck (see Fig. 1). The modified free energy was calculated as the water was dragged through the bottleneck. Results are shown in Fig. 6.

## Discussion

### The AlaThr mutation reduces the bottleneck cross-section area by approximately 30%

The present study supports our previous evaluations of the tight geometry of the Q-chamber entrance (see ref ^21,22^). Namely, we confirm there exists a bottleneck at the entrance of the Q-chamber, and that this bottleneck is too narrow for quinone free passage, as seen in the experimental structures. Table 1 shows that upon mutation the bottleneck cross-section area is further reduced by approximately 25–30%. The reduction in cross-section area is proportional to a reduction in the free volume (and number of states) at the transition state, when the quinone head group passes the bottleneck, leading to an increase in the entropic barrier of quinone passage through the bottleneck. We can estimate the magnitude of the transition state entropy reduction from the reduction of cross-section area. When considering the entropic component of G, we have:

2 $$\Delta {G}_{i}=-{\text{RT}}\mathrm{ln}\left(\frac{{V}_{i}}{{V}_{\text{ref}}}\right)$$

where V_i_ denotes the free volume of species i, and V_ref_ is the free volume available at some reference point along the transition path. V_ref_ can be, for example, the volume inside the Q-chamber. The exact reference does not matter, assuming the reference is the same between the WT and MUT variants. Consider the entrance to the Q-chamber to be a cylinder-like object with face areas A, and width, h. At the transition state, we must reduce the total volume of the bottleneck by excluding that of the quinone headgroup. Assuming the cross section of the latter, A_Q_, we have:

3 $${V}_{i}=\left({A}_{i}-{A}_{Q}\right)h$$

To compare the WT and MUT, we define the entropy difference, $\mathrm{\Delta \Delta }G$, as $\mathrm{\Delta \Delta }G\equiv\Delta {G}_{\text{MUT}}-\Delta {G}_{WT}$. Assuming *h* to be equal in both WT and MUT, we have:

4 $$\mathrm{\Delta \Delta }G=-{\text{RT}}\mathrm{ln}\left(\frac{{A}_{\text{MUT}}-{A}_{Q}}{{A}_{\text{WT}}-{A}_{Q}}\right)$$

It is seen that since the areas involved (A_i_–A_Q_) are within the error of A_i_, the estimates of ΔΔG via geometry analysis are very inaccurate and can serve only as a qualitative indicator; we therefore will have another dynamic method to evaluate entropy changes. Nevertheless, it is interesting to see how different methods compare and we continue the above approach. Using values of our crystal structure bottleneck areas from Table 1, and approximating A_Q_ ≈ 12–14 Å^2^, we see, ΔΔG ≈ 3–5 kJ mol^−1^. (A description of how we arrived upon this approximation, see SI, section “Estimation of A_Q._) It is clear this is a very rough estimate of the barrier, as the cross-sectional area appropriate for bottleneck passage is difficult to define accurately. In fact, 12–14 Å^2^ is a reasonable underestimation of A_Q_ which returns a lower bound on $\mathrm{\Delta \Delta }G$. On the other hand, A_Q_ ≈ 14–15 Å^2^ could as well be a possible estimate, leading to an estimation of ΔΔG ≈ 5–10 kJ mol^−1^. In any case, we anticipate the AlaThr MUT introduces a non-negligible entropic barrier increase.

**Figure 7:**
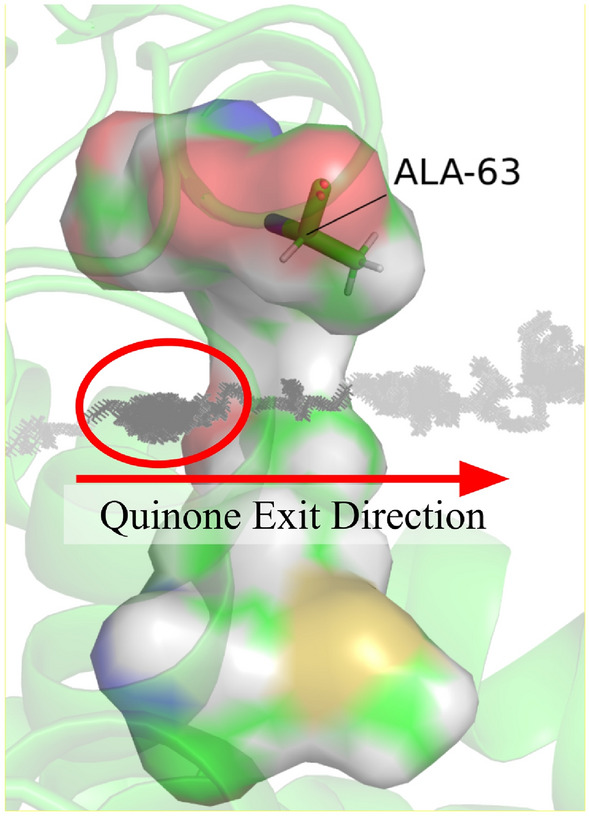
Demonstration of “clustering” in a representative steered MD simulation. A side profile of the bottleneck is rendered above, with the alanine 63 located at the top. Each point represents the position of the head group center of mass when the head group is slowly pulled through the bottleneck.

To further characterize and visualize this entropic barrier, we found it useful to plot the position of the center of mass of the quinone head group during our steered MD simulations. The entropic barriers became easy to identify as “clusters” of points along the pulling trajectory, when the quinone head group is searching for a right orientation to enter the constrained bottleneck, as the passage at the transition state requires almost key and lock match. This search process in advancing to a more constrained geometry shows up as clusters of trajectory points that form in the basins of energetic barriers. Thus, we interpret the trajectory clusters as an indication that the system needing more time to find the correct key and lock match to allow the passage. Therefore, the more points in a cluster, there exists less states that permit the passage of the head group over the barrier. Figure 7 shows such cluster of points along the pulling trajectory.

The number of points within each cluster is related to the (relative) number of states the quinone head group passes through (explores) in a given position along the trajectory. Recalling that entropy is defined as

5 $$S={\text{R}}\mathrm{ln}\left(\Omega \right)$$

where Ω is the number of microstates, we can calculate $\mathrm{\Delta \Delta }G$ via:

6 $$\mathrm{\Delta \Delta }G={\text{RT}}\mathrm{ln}\left(\frac{{\Omega }_{\text{MUT}}}{{\Omega }_{\text{WT}}}\right)$$

**Figure 8:**
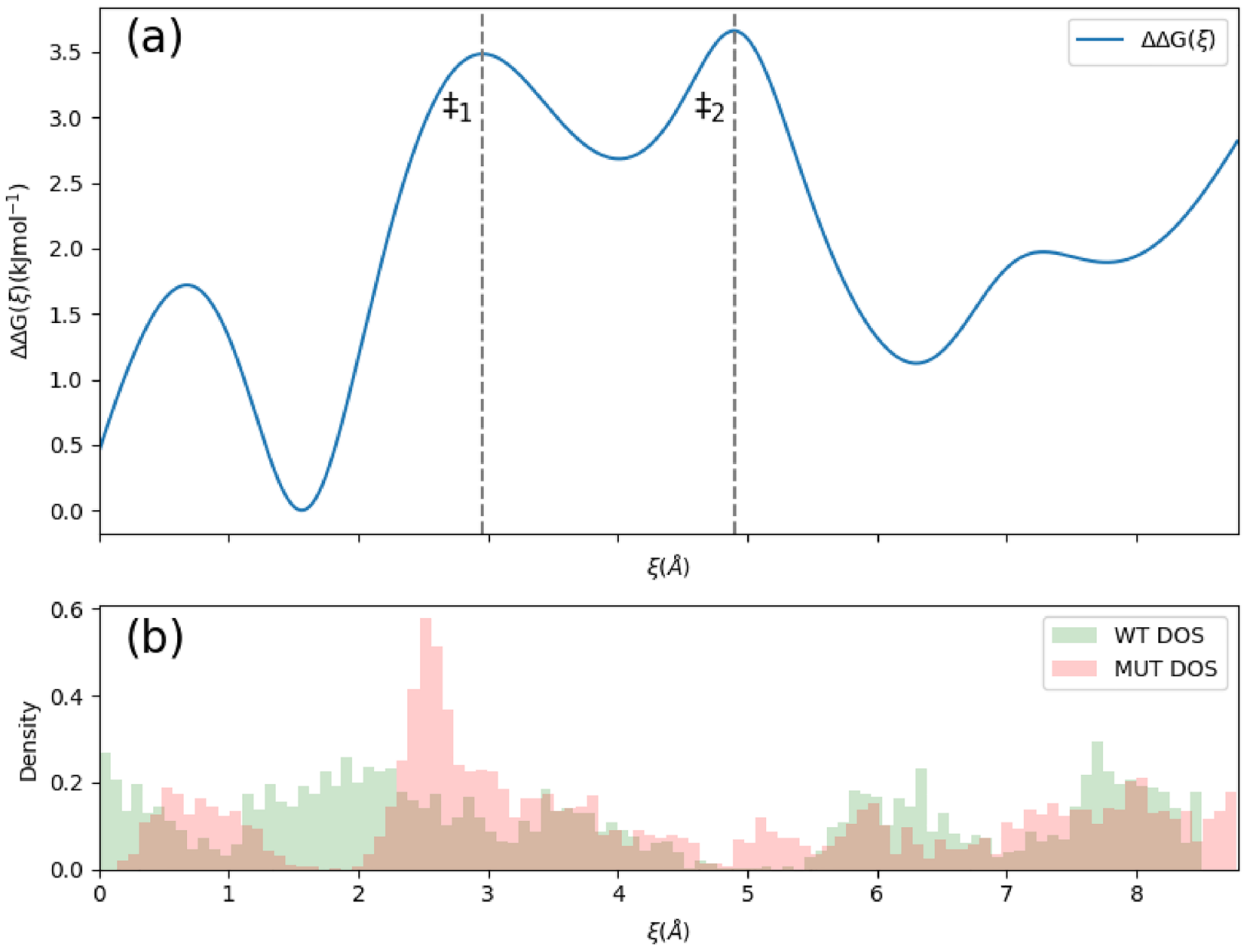
Graph of $\mathrm{\Delta \Delta }G\left(\xi \right)$ (**a**, top), and DOS for both the WT and MUT variants (**b**, bottom). ξ is the same spline used for free energy calculation in Fig. 4. The DOS are approximated by projecting the position of the head group during a steered MD simulation onto ξ. There exist two barriers; the first peak is due to the head group’s entry to the bottleneck, and the second peak is due to the head group’s exit.

By recording the position of the head group center of mass with respect to its displacement through the bottleneck, we can use a Gaussian kernel density estimator to obtain a crude approximation of the density of states (DOS), $\rho \left(\xi \right)$, with respect to our reaction coordinate. Given, $\Omega \left(\xi \right)=\int \rho \left(\xi \right)d\xi$, we obtain $\mathrm{\Delta \Delta }G$ by integrating over ${\rho }_{\text{WT}}\left(\xi \right)$ and ${\rho }_{\text{MUT}}\left(\xi \right)$, and then taking the logarithm of their ratio. Discretizing these equations, $\Omega \left(\xi \right)\approx \sum \rho \left(\xi \right)\Delta \xi$, which simplifies to counting the number of states (points) within a cluster along an interval $\Delta \xi$. Equation 6 can then be used to estimate $\mathrm{\Delta \Delta }G$ . Results are shown in Fig. 8.

We see $\mathrm{\Delta \Delta }G\approx 3.5$ kJ mol^−1^ at ‡^1^, which agrees in order of magnitude with our previous estimation. Additionally, we see there are two peaks in $\mathrm{\Delta \Delta }G$ of the same magnitude. The first peak is due to the head group’s entry to the bottleneck, and the second peak is due to the head group’s exit, which would correspond to the barrier basin in the reverse process for the head group entering the bottleneck from the membrane.

### The bottleneck and head group experience non-negligible deformation during quinone passage

In panels (a) and (c) of Fig. 2, there exists non-negligible deformation of the quinone head group and bottleneck during quinone passage. When the bottleneck is strongly restrained, we observe there exists a 30 kJ mol^−1^ deformation barrier, resulting from the deformation of the quinone head group from its equilibrium position. This barrier is present in both the wild type and AlaThr variants. Previously, we proposed all energetic barriers of 30 kJ mol^−1^ or less should be considered open (which can be overcome with thermal fluctuations within the “squeeze-in” model), whereas barriers in gross excess of this threshold are closed (see ref ^22^). Our results confirm the bottleneck is very tight and essentially closed in both the wild type and AlaThr MUT, requiring a special mechanism such as squeeze-in mechanism of Ref. 22. However, when unrestrained, the bottleneck cross-section area increases to allow quinone head group easier passage (Fig. 2b and d). We see ${A}_{\text{WT}}^{max}=27.2$ Å^2^ and ${A}_{\text{MUT}}^{max}=19.4$ Å^2^. These maximal areas each correspond to a 30% increase from the rigid/crystal bottleneck cross-section area. Notably, the unrestrained simulations do not feature significant head group deformation. We should mention that the total cross-section area does not capture all nuances of the tight passage through the bottleneck. It neglects the width of the bottleneck which lends the head group an additional degree of freedom. As a result, despite the quinone head group and bottleneck having a kind of key-and-lock match, a 30% decrease in bottleneck cross-section area does not prevent quinone passage through the bottleneck, but it does lead to a severe disruption. Thus, our estimates can only serve a rough semi-quantitative purpose.

### Quinone transport is severely disrupted by AlaThr MUT

The bottleneck passage rate of a quinone molecule can be estimated by the transition state theory. The rate of a single barrier crossing of the quinone head group is given by,

7 $$k\approx \frac{{D}_{Q}}{{L}_{\circ }^{2}}{e}^{-\frac{\Delta G}{\text{RT}}}$$

where D_Q_ denotes the diffusion coefficient of quinone in membrane environment, D_Q_ ~ 10^–8^ cm^2^s^−1^, and L_o_ denotes the characteristic length of the barrier width. To determine the impedance effect on the rate of quinone passage by the mutation, we will use the free energy barrier presented in the interval ξ ~ [− 0.5,3]. We see G_WT_ ≈ 28 kJ mol^−1^, and G_MUT_ ≈ 33 kJ mol^−1^. From Eq. 7, we see,

8 $$\frac{{k}_{\text{WT}}}{{k}_{\text{MUT}}}={e}^{\frac{\mathrm{\Delta \Delta }G}{\text{RT}}}\approx 7.5$$

We see, roughly the rate of quinone passage across the bottleneck is almost five- to tenfold slower in the AlaThr MUT compared to WT.

Additionally, we estimated earlier ΔΔG ≈ 5 kJ mol^−1^, which agrees in order of magnitude with our previous predictions. Although 5 kJ mol^−1^ is not an objectively large amount of energy (for scale, the strength of water hydrogen bond is ~ 20 kJ mol^−1^), it is a reasonably large energetic barrier to arise from entropy alone. Obviously, there exists a non-negligible entropic barrier preventing quinone’s free passage through the bottleneck. The rate of quinone passage is slower in the AlaThr MUT due to entropy reduction indicated by the decrease in cross-section area of the bottleneck. Although the total area cross section does not capture all the nuances of tight passage of irregular-shaped quinone through the irregular shaped bottleneck (Fig. 1), which requires a kind of key and lock match, qualitatively, the reduction of entropy at the transition state is a clear indication of a more difficult (slower) passage of MUT through the bottleneck.

Slowing quinone passage five- to tenfold may have severe biochemical consequences. Slowed quinone passage can lead to slowing down electron transport in complex I, effectively inhibiting the quinone site. It has been well established inhibition of the quinone site accelerates ROS production due to inhibition of forward electron transfer at the FMN site^12,31,34–37^. This could lead to electron back up at the upstream FMN site in complex I, which may become over-reduced and therefore give rise to superoxide production and related oxidative stress in the cell. Although our work cannot quantify the severity of the defect, qualitatively, ROS production in the AlaThr MUT can be related to the decreased cross-sectional area of the bottleneck and related slowed down quinone binding kinetics. Previous experimental work with the complex I of *Paracoccus denitrificans* has determined kinetic parameters for Q_1_, Q_2_, and Q_10_ binding for both the wild type complex I, and of the AlaThr MUT^38^. It was shown the AlaThr MUT lead to an increase in the K_M_ and decrease in the V_max_ of quinone reduction activity. This is a clear indication of slowed quinone binding kinetics due to the AlaThr MUT, matching our model’s prediction. Additional experiments have demonstrated electron transfer activity is reduced by 60–80% in MUT^31^. Assuming the entirety of electron transfer decrease is due to the slowed quinone binding kinetics, slowing quinone binding five- to tenfold leads to electron transfer being decreased by 80%-90%. We see our model and calculations roughly agree with experiment, though our model overestimates the decrease in electron transfer activity.

Our model likely overestimates the decrease in electron transfer activity because we studied *T. Thermophilus* CI, whereas experimental studies of this mutation have primarily focused on human CI. The human CI bottleneck is slightly less narrow that of *T. Thermophilus* (A_WT_ = 22.94 ± 0.01 Å^2^, see Fig. S4.) If we perform the same calculation of ΔΔG as we did in discussion Sect. 1 and assuming the human CI bottleneck cross-section area is reduced by 30%, we see ΔΔG ~ 2–5 kJ mol^−1^ for A_Q_ 12–14 Å^2^. This rough estimate of ΔΔG corresponds to a 60%-80% decrease in electron transfer activity. All analysis involving the cross-section area of the bottleneck should be taken as semi-quantitative as it neglects several nuances involved in the quinone head group passage. Our findings provide evidence decreased electron transfer activity is related almost entirely to the reduced cross-section area of the bottleneck.

### Water is dynamic in wild type complex I

Comparing the free energy profiles in Fig. 6, we see AlaThr MUT presents an additional 15 kJ mol^−1^ energetic barrier to the passage of water through the bottleneck (when quinone is bound). Using Eq. 7 with the energetic barrier for ξ ~ [0.5,6], we see:

9 $$\frac{{k}_{\text{WT}}}{{k}_{\text{MUT}}}\approx 400$$

That is, the rate of water passage (i.e. exchange) through the bottleneck is approximately 400 times slower in AlaThr MUT than in the wild type. To put a time scale to this process, we again use transition state theory to estimate the rate of water dissociation. Taking for water in the membrane environment D_HOH_ ~ 10^–5^–10^–6^ cm^2^ s^−1^, and assuming our characteristic length (barrier width) to be approximately 3 Å, from Eq. 8 we obtain ${\tau }_{\text{WT}}={k}_{\text{WT}}^{-1}=0.1-1.0$ μs. It appears this timescale is a bit overestimated. Teixeira et. al. found water to diffuse through the bottleneck (when quinone tail is present) on the sub-microsecond timescale^25^. We therefore ran a small set of MD simulations with water placed inside the bottleneck and allowed the water to freely diffuse. We observed water diffused on sub-microsecond timescale, agreeing with Teixeira et. al. (see SI Fig. 2). Which could indicate that the taken diffusion coefficient 10^–5^–10^–6^ cm^2^ s^−1^ for water is underestimated.

Regardless of whether water passage is on the sub-microsecond or microsecond timescale in the wild type variant, it is still orders below that of complex I’s catalytic cycle (~ ms). As a result, we propose water is free to pass across the bottleneck when quinine is bound. If, in reality, water passage across the bottleneck is at the microsecond time scale, then a factor of 400 in the AlaThr variant will push water exchange to the low millisecond timescale, which is the same order of magnitude as complex I’s timescale. As a result, water exchange in AlaThr MUT may be impossible when quinone is bound, which means it can severely affect (slow down) the overall binding kinetics. If water exchange through the bottleneck is critical for quinone binding, slowing this process may be another avenue of bioenergetic defect, potentially leading to LHON symptoms.

## Conclusions

We confirmed there exists a geometric bottleneck at the entrance to the Q-chamber. The AlaThr MUT results in a further decrease in the cross-section area of the bottleneck, leading to transition state entropy reduction and related increase of the transition state barrier by 5 kJ mol^−1^. We calculated the entropy reduction by three independent methods, and all three agree within an order of magnitude. We show this entropy reduction results in quinone passage through the bottleneck to be slowed down five- to tenfold in MUT relative to the wild type. Additionally, we showed water is dynamic across the bottleneck in the wild type variant, but water exchange is severely disrupted in the MUT variant. The function of the narrow bottleneck remains obscure; in no other quinine-binding sites in ETC does a narrow bottleneck exist except in complex I.

We conclude that quinone passage is slowed in the AlaThr MUT. The tight bottleneck at the entrance of the Q-chamber in WT complex I is the cause of the severity of the AlaThr MUT. In the mutant, the already tight bottleneck becomes even tighter, severely disrupting quinone binding kinetics and thereby perturbing ETC. We propose that slowed down quinone passage leads to increased ROS production by complex I, which potentially can lead to symptoms presented in LHON patients. The measurements of quinone binding kinetics to complex I in both the WT and MUT variants and ROS production should further clarify the mechanism.

## Materials and methods

### MD simulation details

All MD simulations were performed in Gromacs^39^ with the CHARMM36^39,40^ force field. We modified the residue topology of neutral arginine (ARGN) because the atom-types used in the native distribution do not have the correct valency. This was resolved by using the correct atom-types. Our simulations were performed using only subunits forming the Q-chamber (Nqo4, Nqo6, and Nqo8) in vacuum in the NVT ensemble. Our protein topology was generated using the pdb2gmx tool in Gromacs. All Q_i_ topologies were generated using the CGenFF webserver^41^. System was electrically neutral, neutralized through protonation/deprotonation of charged residues. During energy minimization and equilibration, protein atoms were restrained to preserve as close to the crystal structure as possible. Equilibration simulations were 100 ps long. Protein backbone atoms were restrained with a force constant of 50 kJ mol^−1^ Å^−2^, and protein sidechain atoms were restrained using a force constant of 25 kJ mol^−1^ Å^−2^. Quinone atoms were restrained using a force constant of 10 kJ mol^−1^ Å^−2^. The PDB structure was used to define the initial/equilibrium positions of atoms. Restraints were used during equilibration and production MD. All simulations used a time step of 0.002 ps with a leap-frog stochastic dynamics integrator. We used a velocity rescale thermostat^42^ with a temperature coupling time constant of 0.1 ps. Short-range Coulomb and van der Waals interactions were cut-off at 1.2 nm. Particle Mesh Ewald summation was used to handle long-range electrostatics. (For additional details, see related Figs. S5–S8 and S12–S15 in SI.)

During our steered MD simulations, Q_1_ was placed near the 2’ position of ref ^33^ in both the WT and MUT variants (see Fig. S16 in SI for details). Q_1_ was pulled by end carbon with a rate of 0.04 Åps^−1^ with a force of 25 kJ mol^−1^ Å^-2^; the passage of some 12 angstroms distance required ~ 300 ps. Multiple pulling trajectories were collected. To construct the MUT variant, Alanine 63 in Fig. 1 was substituted with a threonine in silico. The substitution was performed using the mutagenesis tool native to PyMOL.

### Umbrella sampling protocol

For all US windows, the Q1 head group was harmonically restrained with a force constant of 20 kJ mol^−1^ Å^−2^. The head group was restrained with a zero-mass, zero-charge, virtual site placed at the geometric center of the head group ring. Windows were evenly spaced, with approximately 0.3 Å spacing between adjacent windows, and sampled for 5 ns. Our reaction coordinate was constructed as follows:

- One representative MD pulling trajectory was used to track the position of the head group in space
- A spline was fit to the positions to approximate the path the head group followed during the steered MD simulation
- During US, the displacement of the virtual site relative to its initial position was measured, and then projected onto the spline

Windows were joined via umbrella integration. To analyze the energy, the trajectories were rerun with the models constrained to the quinone head group. The average nonbonded energy between the head group and protein was calculated for all points along the reaction coordinate, along with their standard deviation. A spline was fit to the average and subtracted from the free energy profile.

The above procedure is the same for our water US simulations, except with a force constant of 80 kJ mol^−1^ Å^−2^ applied to the water oxygen.

### Supplementary Information

Supplementary Information.

## Data Availability

All data generated or analyzed during this study are included in this published article and its Supplementary Information (SI) file.
