# Supplementary material for: Mutation at the entrance of the quinone cavity severely disrupts quinone binding in respiratory complex I
Source: Sci Rep. 2023 Nov 21;13:20413. doi: 10.1038/s41598-023-47314-2 (PMC10663621; doi:10.1038/s41598-023-47314-2)
Supplement: Supplementary file 1 — Supplementary Information. [file 41598_2023_47314_MOESM1_ESM.docx]

**Supplementary Information for**

**Mutation at the entrance of the quinone cavity severely disrupts quinone binding in respiratory complex I**

Jason Tae Yi, Panyue Wang, and Alexei A. Stuchebrukhov*

**1. Novel cross-section area algorithm**

1. **Algorithm**

The following algorithm was developed to accept a bottleneck structure file and return the approximate cross-section area of the bottleneck. In principle, this algorithm can be used for any structure (or dataset of points in **R**^3^), so long as the structure is toroidal.

Given a structure file, perform the following:

- 1. Calculate the solvent accessible surface (SAS, or Connolly surface^1, 2^)
     1. Returns point cloud of the surface
  2. With point cloud, perform principal component analysis (PCA)
  3. Project point cloud onto plane normal to the PCA component with least variance
  4. Rotate plane into the xy-plane^3^, and calculate the alpha shape
     1. (at least) two polygons will be returned, an outer polygon representing the perimeter of the bottleneck, and an inner polygon representing the perimeter/cross-section of the bottleneck
  5. Calculate the surface area of the interior alpha shape via the Shoelace method^4^

The above algorithm was implemented in Python. SAS calculations were performed using PyMOL^5^. The numpy^6^, scipy^7^, and scikit^8^ packages were used to aid in numerical calculations.

1. **Limitations**

Refs ^2, 9^ discuss how displacement of specific atoms can lead to large changes to the solvent accessible surface. As described by Grant et. al.: SAS has “unpleasant characteristic [that] the infinitesimal movement of a single atom can produce large surface changes.” As a result, we do not claim this algorithm gives an accurate result, but it does provide a good approximation as to what the true cross-section is, and has, proved to provide solid qualitative and quantitative descriptions of our system.

A second limitation is the choice of projection vector. The PCA component of least variance of a torus (and torus-shaped data) will, in general, be qualitatively normal to the opening. As a result, PCA provides a simple and efficient method to calculate a good candidate vector. A rigorous statement will not be made about how accurate this vector is for capturing the true minimum cross-section area.

**2. Qualitative rendering of the transition state entropy reduction**


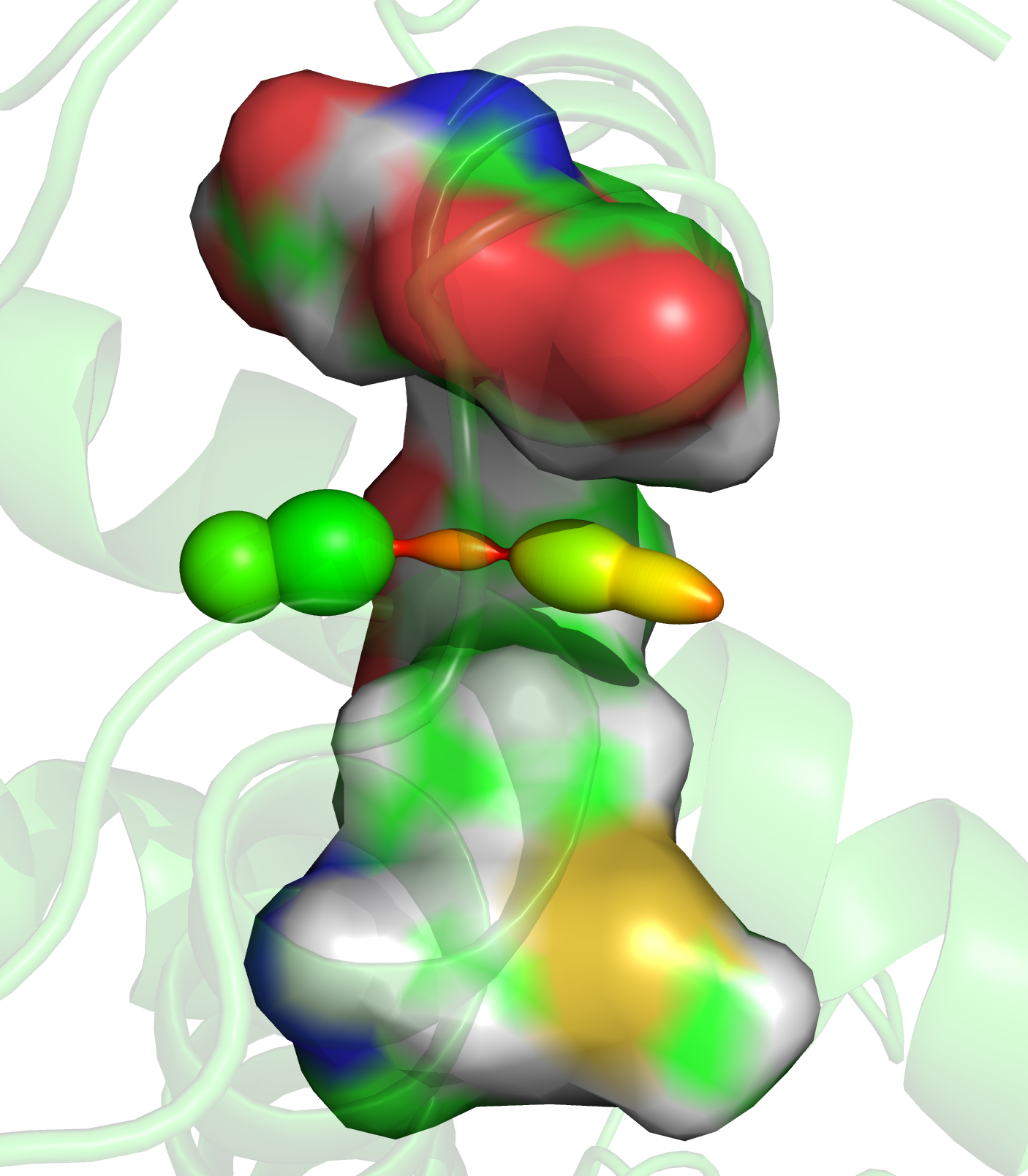


**Figure S1.** $\Delta\Delta G$ calculated in figure 8 visualized in PyMOL. Smaller radii indicate larger $\Delta\Delta G$, meaning these are sites of large entropy reduction.

**3. Water is dynamic across bottleneck**


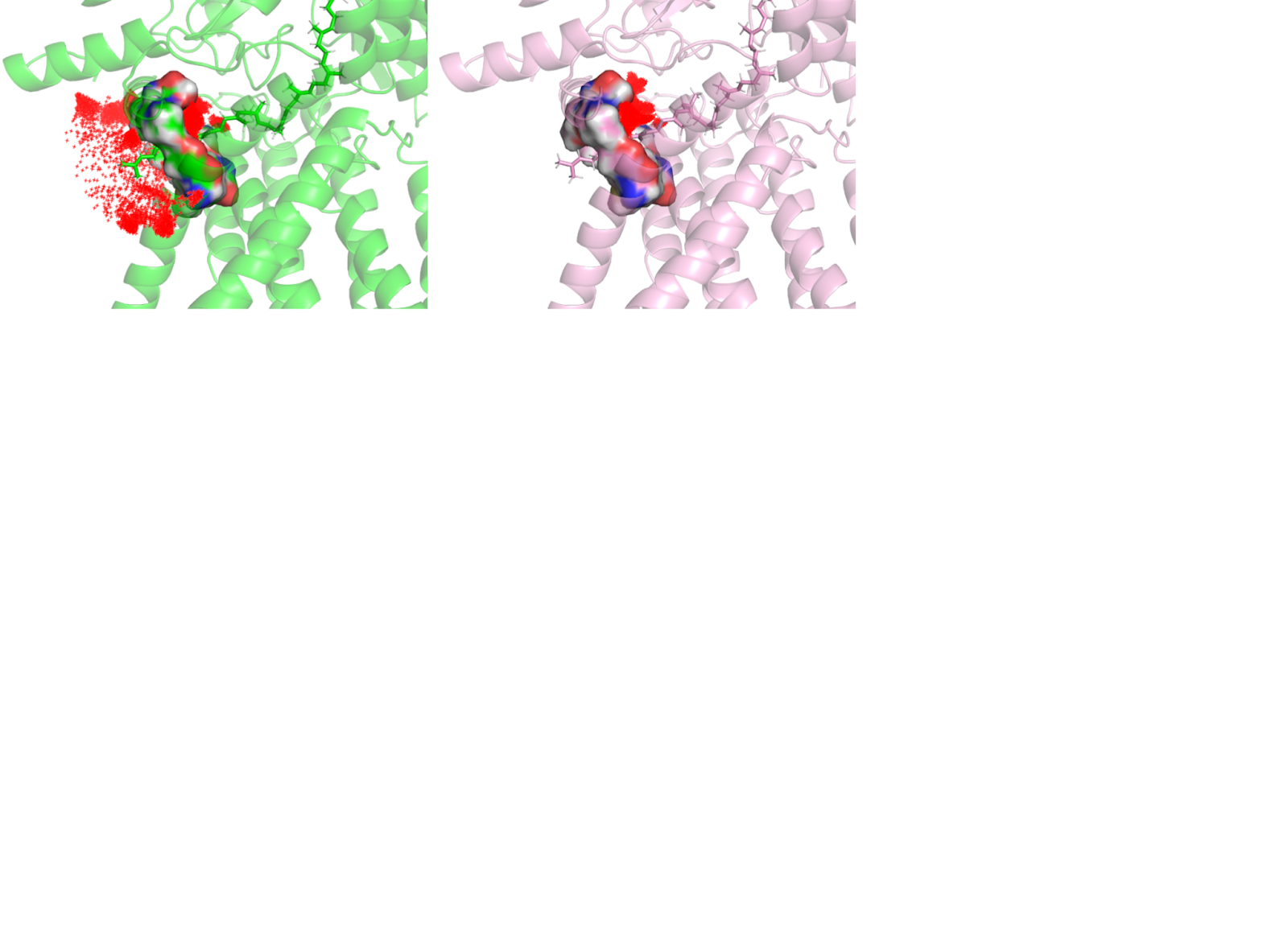


**Figure S2.** Visualization of water molecule trajectories during 30, 20 ns MD simulation in the WT (left) and MUT (right) variants. A flat-bottom restraint was imposed to prevent water from diffusing far from the bottleneck. Water was not observed diffusing through the bottleneck in the MUT variant. This does not support or refute claims that water is not dynamic in the MUT case, but does illustrate water dynamics are different between the two variants.

**4. Comparisons to human complex I bottleneck**

The entrance to the human complex I Q-chamber presents a geometric bottleneck. The cross-section area and composition. As a result, we believe this justifies using the complex I structure of *T. Thermophilus* to draw incite on the impact of the 3460 mtDNA MUT (referred to as AlaThr MUT in main text).


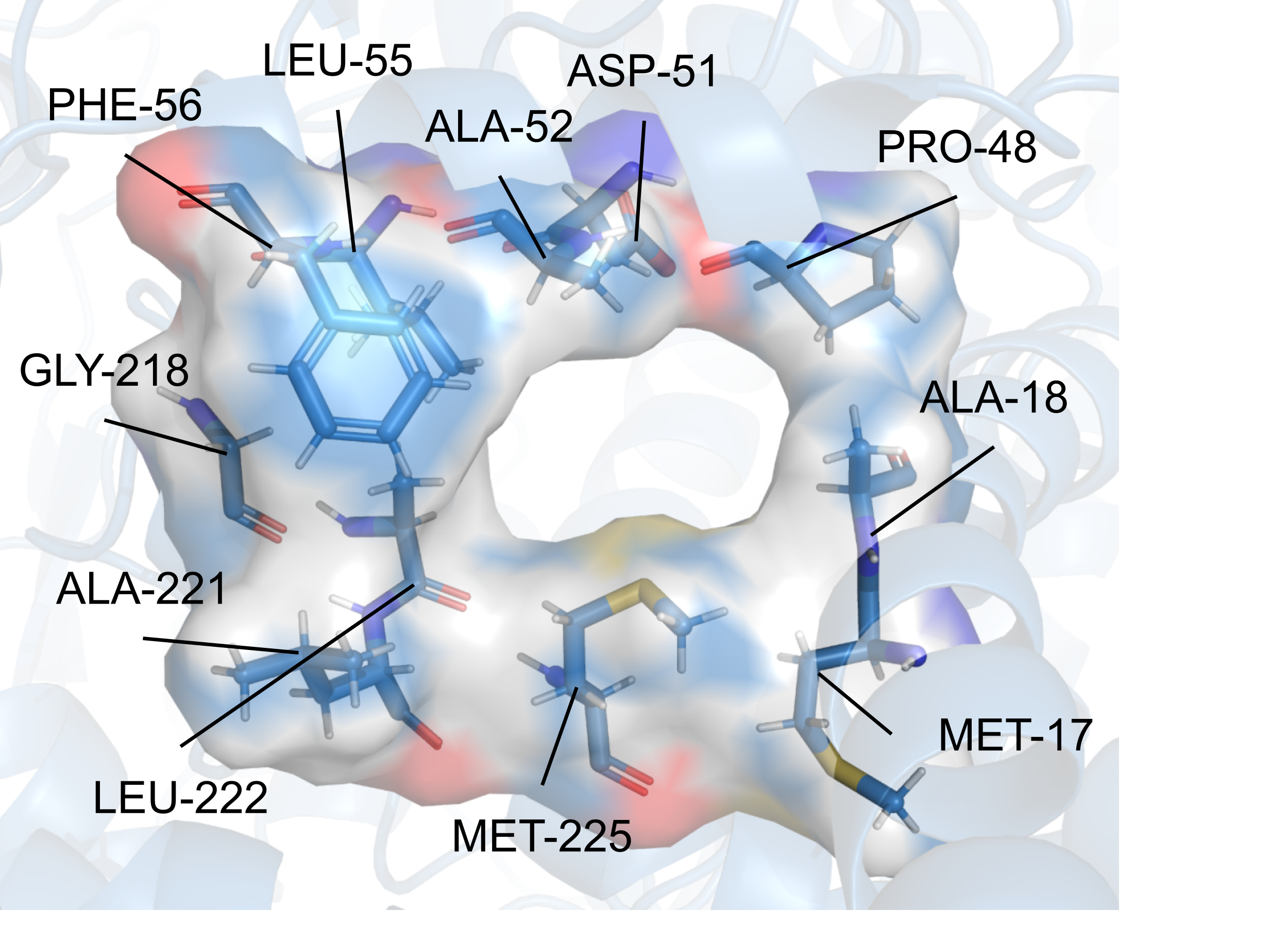


**Figure S3.** Annotated visualization of the human bottleneck (PDB: 5XTD). The bottleneck construction is well conserved between human and T. Thermophilus complex I. Reading clockwise, Ala52, Asp52, Pro48, Ala18, Met225, Leu222, and Ala221 are all conserved.


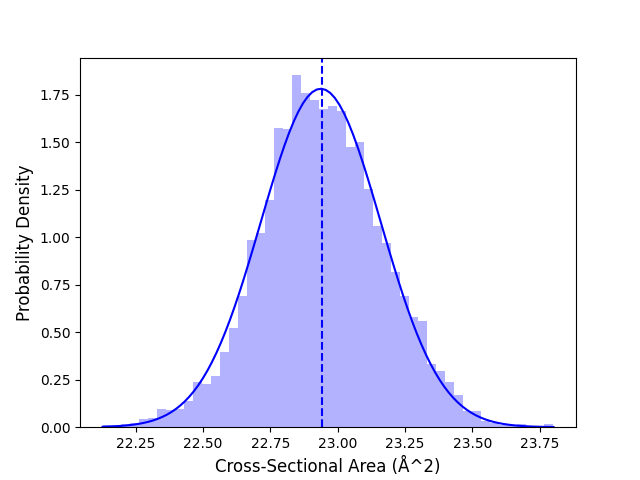


**Figure S4.** Distribution of human bottleneck cross-section area during a 300 ps MD simulation without quinone bound, and all atoms in the bottleneck strongly restrained as in the PDB. The human bottleneck has a cross-section area of 22.94 ± 0.01 Å^2^ in the crystal structure.

**5.** **Time series of cross-section areas and quinone head group deformation**

**
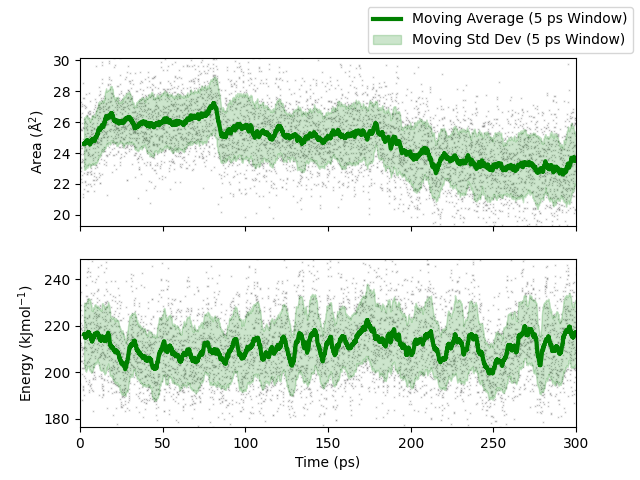
**

**Figure S5.** Time series of bottleneck cross-section area and quinone head group deformation energy of the wild type variant in the relaxed simulation conditions.

**
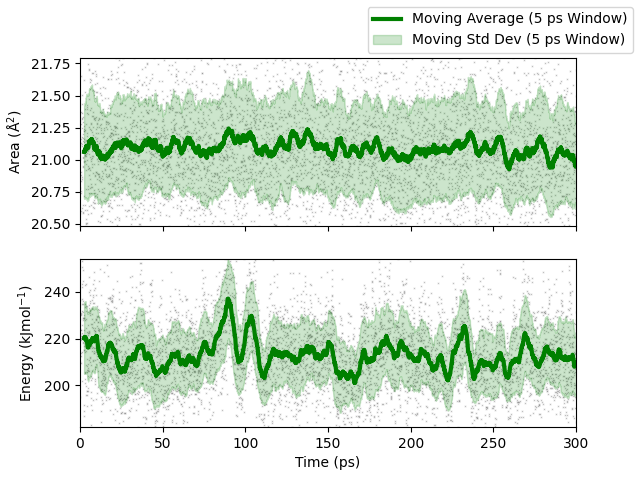
**

**Figure S6.** Time series of bottleneck cross-section area and quinone head group deformation energy of the wild type variant in the rigid simulation conditions.

**
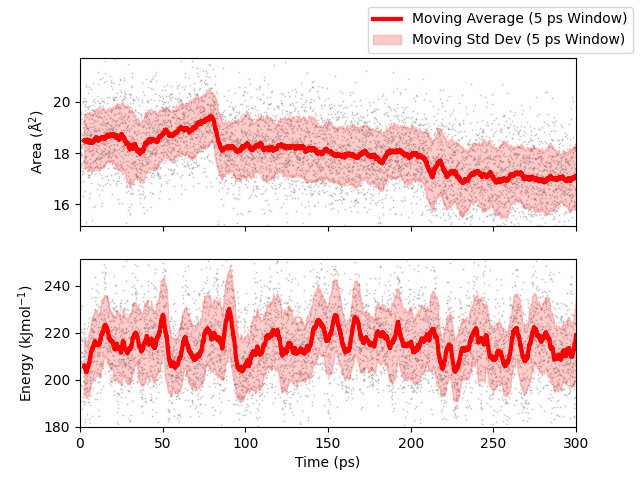
**

**Figure S7.** Time series of bottleneck cross-section area and quinone head group deformation energy of the AlaThr MUT in the relaxed simulation conditions.

**
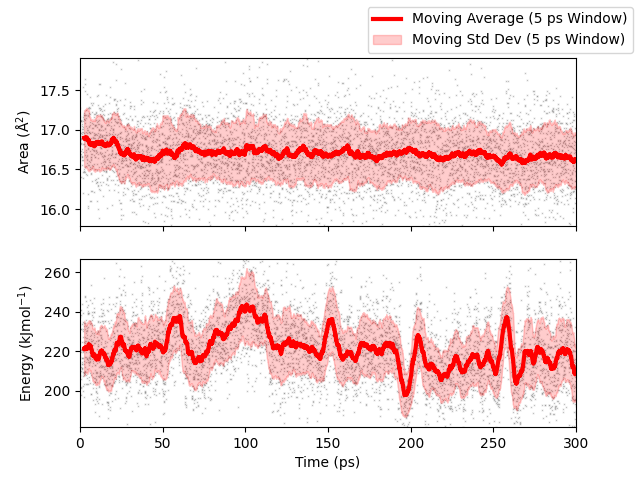
**

**Figure S8.** Time series of bottleneck cross-section area and quinone head group deformation energy of the AlaThr MUT in the rigid simulation conditions.

**6. Estimation of A­_Q_**

To obtain a rough estimation of the cross-section area of quinone head group, we first simplify the quinone head group to be represented solely by the six-member ring. Additionally, we will assume each carbon-carbon bond length is 1.4 Å, and the Van Der Waals radius of carbon is 1.7 Å.

Consider placing the benzene ring parallel to the xy-plane (see fig. S9). We first project the benzene ring onto the x-axis (fig S10). Next, we consider only the first quadrant of the benzene ring (fig S11).


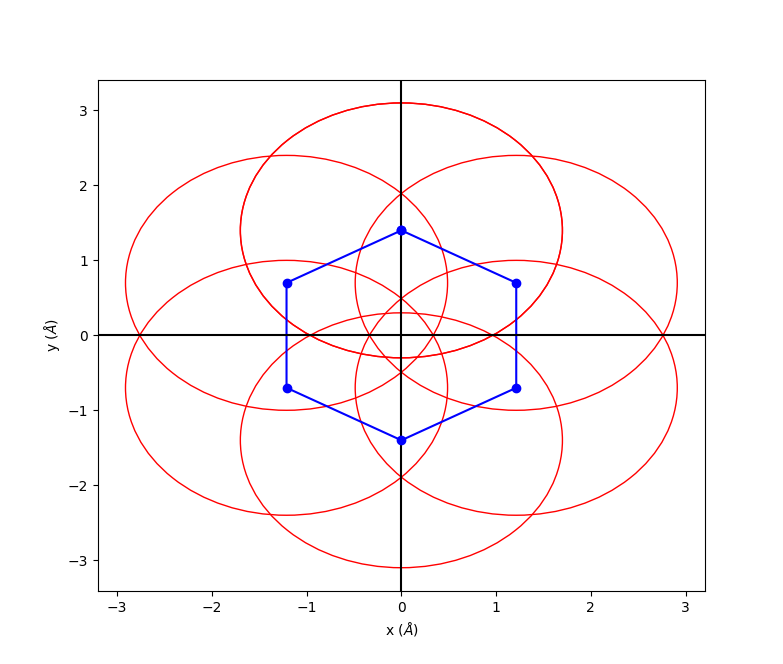


**Figure S9.** Rough illustration of our model benzene ring with carbon-carbon bond length equal to 1.4 Å, and the Van Der Waals radius of carbon equal to 1.7 Å. Blue lines indicate bonds, blue dots indicate atom centers, and red circles indicate atom radii.


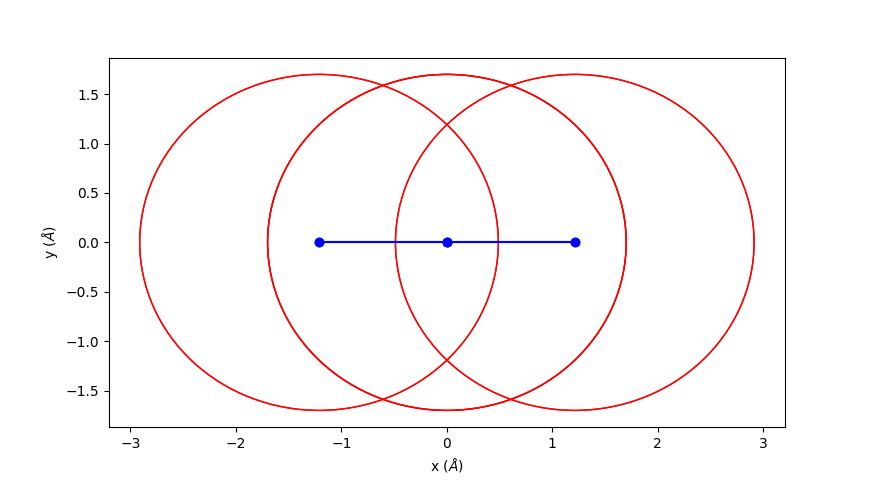


**Figure S10.** Projection of our benzene ring onto the x axis. This is the face of the molecule we will compute the cross-section area of, as this is a rough estimation to the true quinone head group cross-section.


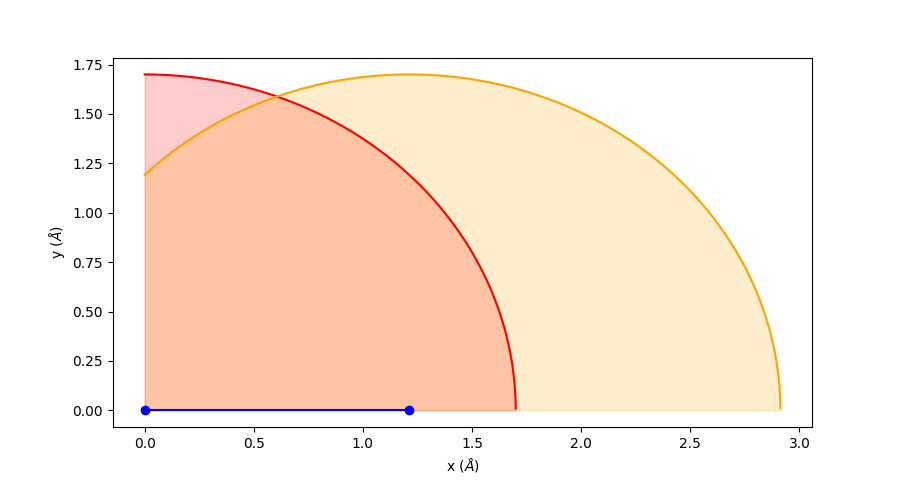


**Figure S11.** First quadrant of figure S6, with areas highlighted. Calculating the total cross-section area of our model system reduces to calculating the area of the above figure, and then multiplying by four.

The red curve in fig. S7 is given by $f\left( x \right)=\sqrt{r_{C}^{2}-x^{2}}$ for $x\in\left[ 0,r_{c} \right]$, where $r_{c}\equiv1.7$ Å. Whereas the orange curve in fig. S7 is given by $g\left( x \right)=\sqrt{r_{C}^{2}-\left( x-\cos\left( \frac{\pi}{6} \right)d_{CC} \right)^{2}}$ for $x\in\left[ 0,r_{C}+\cos\left( \frac{\pi}{6} \right)d_{CC} \right]$. Given forms of $f\left( x \right)$ and $g\left( x \right)$, it is simple to calculate the area of fig. S7. Let $x^{*}$ denote the value of x such that $f\left( x^{*} \right)=g(x^{*})$, and ${\alpha=r}_{C}+\cos\left( \frac{\pi}{6} \right)d_{CC}$, we see A_Q_ is given by,

$A_{Q}=4[\int_{0}^{r_{C}} f\left( x \right)dx+\int_{0}^{a} g\left( x \right)dx-\int_{0}^{x^{*}} g\left( x \right)dx-\int_{x^{*}}^{r_{C}} f\left( x \right)dx]\approx17$ Å^2^

This sets a rough upper bound on the total cross-section area of quinone which passes through the bottleneck. In order to use eq. 4 of the main text, we require A_Q_ to be less than A_MUT_­ (that is, any estimations of A_Q_ should be less than 15.10 Å^2­­^). An even simpler approximation can be used, as well. Approximating the shape in fig S10 as a rectangle with side lengths $2(r_{C}+d_{CC})$ and $2d_{CC}$, we see the cross-section of the benzene molecule is less than 17.4 Å^2^.

17 Å^2^ bounds all estimations of A_Q_ from above. However, eq. 4 can be used to restrain our estimation of A_Q_ further. For eq. 4 to return a reasonable estimation of ΔΔG, we require A_Q_ < A_MUT_. In consideration to our previous work on the subject^10, 11^, we believe there exists a key-and-lock type mechanism for quinone to enter its binding site. Therefore, we anticipate A_Q_ ≈ A_MUT_. We’ve updated our manuscript to approximate A_Q_ ≈ 14-15 Å^2^.

**7. Bottleneck cross-section area in AMBER 94 and OPLS-AA forcefield**

1. **AMBER 94^12^**


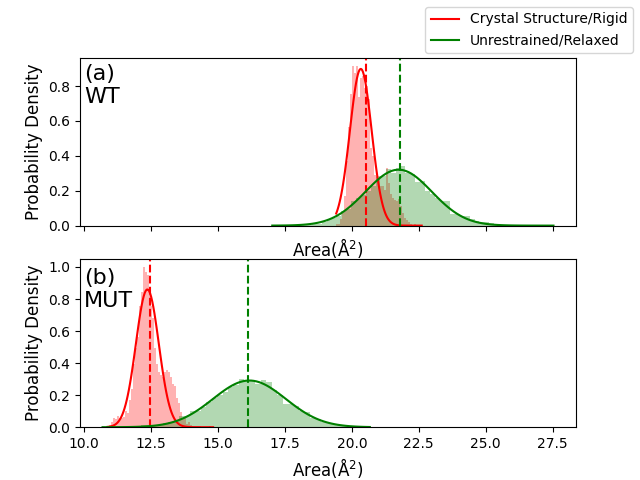


**Figure S12.** The cross-section area distributions of the *T. Thermophilus* the wild type (a, left) and AlaThr (b, right) variant bottlenecks in the rigid (red) and relaxed (green) simulation conditions. Simulations were performed identical to simulations presented in fig. 3 in main text, except the AMBER 94 forcefield was used.

**Table S1.** Summary of bottleneck cross-section area from the distributions plotted in Fig. S12, along with the percent change between the wild type (WT) and the AlaThr (MUT) bottleneck variants.

| **Restraint** | **WT** | **MUT** | **Percent Change** |
| --- | --- | --- | --- |
| **Crystal Structure/Rigid (Å^2^)** | 20.52 ± 0.01 | 12.47 ± 0.01 | -39.2% |
| **Unrestrained/Relaxed (Å^2^)** | 21.80 ± 0.03 | 16.13 ± 0.04 | -26.01% |

Via eq. 4 of the main text, using values for A_MUT_ and A_WT_ from table S1, and estimating A­_Q_ ≈ 11-12 Å^2^, we see, ΔΔG ≈ 5-8 kJmol^-1^.

1. **OPLS-AA^13^**


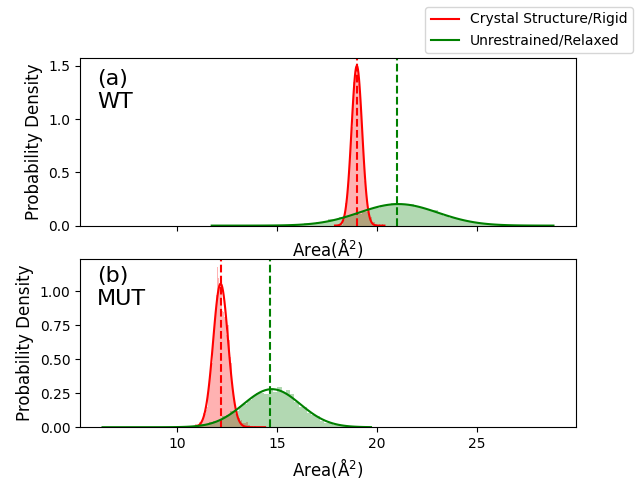


**Figure S13.** The cross-section area distributions of the *T. Thermophilus* the wild type (a, left) and AlaThr (b, right) variant bottlenecks in the rigid (red) and relaxed (green) simulation conditions. Simulations were performed identical to simulations presented in fig. 3 in main text, except the OPLS-AA forcefield was used.

**Table S2.** Summary of bottleneck cross-section area from the distributions plotted in Fig. S13, along with the percent change between the wild type (WT) and the AlaThr (MUT) bottleneck variants.

| **Restraint** | **WT** | **MUT** | **Percent Change** |
| --- | --- | --- | --- |
| **Crystal Structure/Rigid (Å^2^)** | 19.01 ± 0.01 | 12.20 ± 0.01 | -35.8% |
| **Unrestrained/Relaxed (Å^2^)** | 21.00 ± 0.05 | 14.67 ± 0.04 | -30.1% |

Via eq. 4 of the main text, using values for A_MUT_ and A_WT_ from table S1, and estimating A­_Q_ ≈ 11-12 Å^2^, we see, ΔΔG ≈ 5-10 kJmol^-1^.

**8. Nonbonded potential and average force along reaction coordinate**


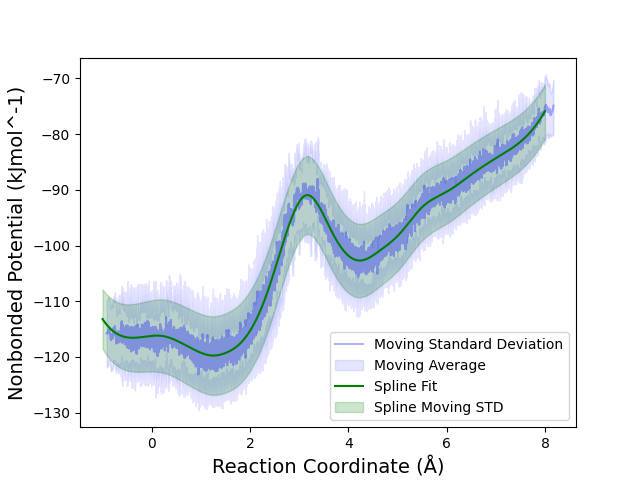


**Figure S14.** Plot of nonbonded potential between the quinone headgroup and protein with respect to the reaction coordinate.


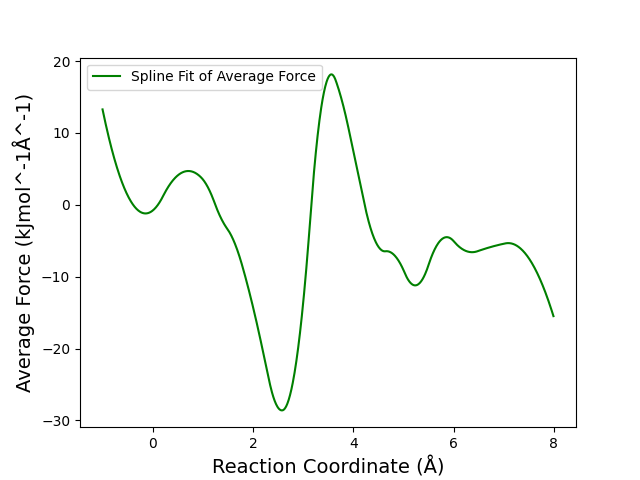


**Figure S15.** Plot of average force on the quinone headgroup when passing through the bottleneck with respect to the reaction coordinate. This plot was obtained through numerically differentiating the potential in S14.

**9. Initial position of Q1 in pulling simulations**


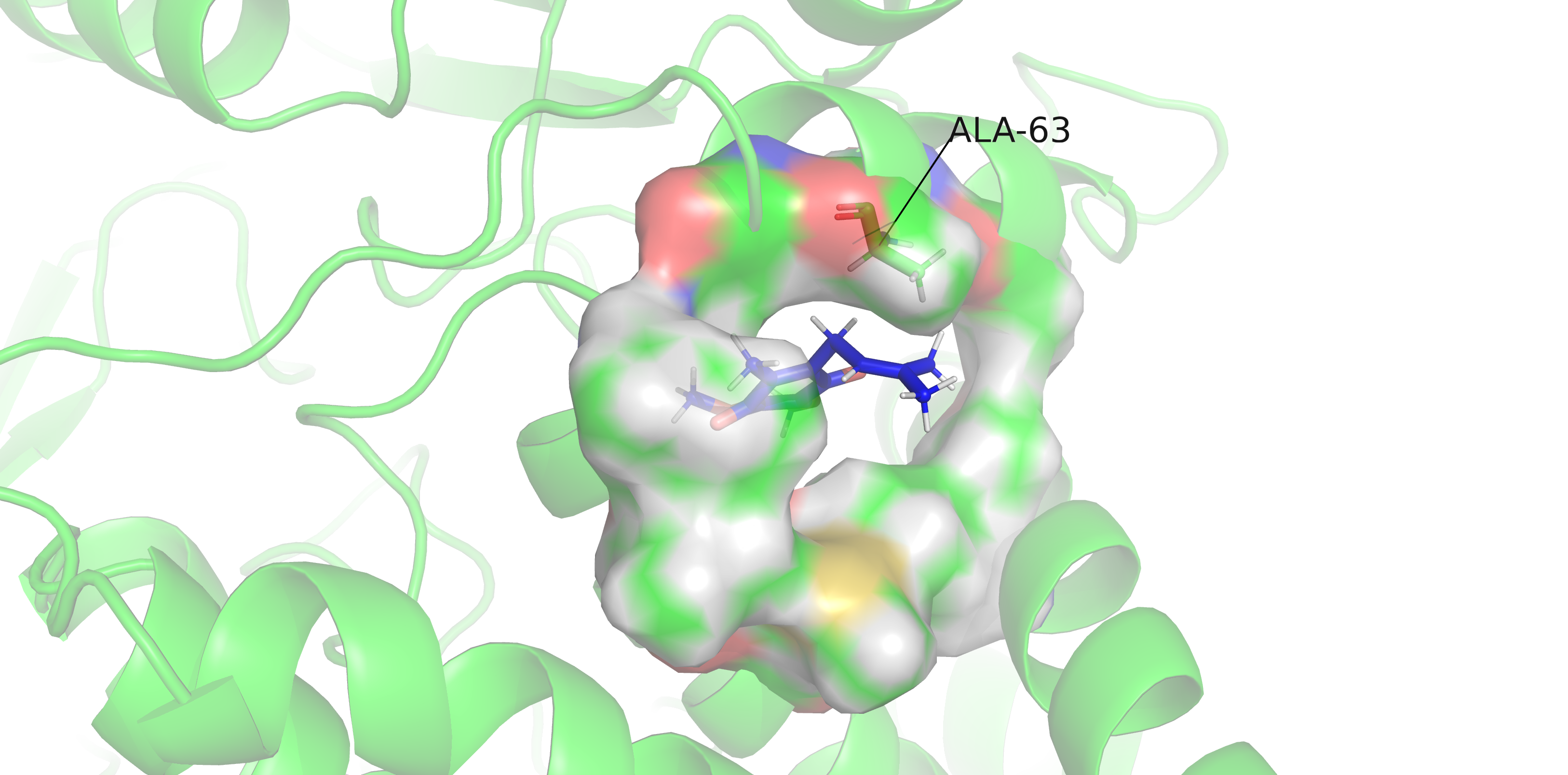


**Figure S16.** Q1 in the Q-chamber near initial position for all steered MD simulations.

**SI References**

(1) Connolly, M. L. Solvent-accessible surfaces of proteins and nucleic acids. *Science* **1983**, *221* (4612), 709-713. DOI: 10.1126/science.6879170 From NLM Medline.

(2) Grant, J.; Pickup, B.; Nicholls, A. A smooth permittivity function for Poisson-Boltzmann solvation methods. *Journal of Computational Chemistry* **2001**, *22*, 608-640. DOI: 10.1002/jcc.1032.

(3) Dai, J. S. Euler–Rodrigues formula variations, quaternion conjugation and intrinsic connections. *Mechanism and Machine Theory* **2015**, *92*, 144-152. DOI: <https://doi.org/10.1016/j.mechmachtheory.2015.03.004>.

(4) Braden, B. The Surveyor's Area Formula. *The College Mathematics Journal* **1986**, *17* (4), 326-337. DOI: 10.1080/07468342.1986.11972974.

(5) Schrodinger, LLC. The PyMOL Molecular Graphics System, Version 1.8. 2015.

(6) Harris, C. R.; Millman, K. J.; van der Walt, S. J.; Gommers, R.; Virtanen, P.; Cournapeau, D.; Wieser, E.; Taylor, J.; Berg, S.; Smith, N. J.; et al. Array programming with NumPy. *Nature* **2020**, *585* (7825), 357-362. DOI: 10.1038/s41586-020-2649-2 From NLM Medline.

(7) Virtanen, P.; Gommers, R.; Oliphant, T. E.; Haberland, M.; Reddy, T.; Cournapeau, D.; Burovski, E.; Peterson, P.; Weckesser, W.; Bright, J.; et al. SciPy 1.0: fundamental algorithms for scientific computing in Python. *Nat Methods* **2020**, *17* (3), 261-272. DOI: 10.1038/s41592-019-0686-2 From NLM Medline.

(8) Pedregosa, F.; Varoquaux, G.; Gramfort, A.; Michel, V.; Thirion, B.; Grisel, O.; Blondel, M.; Prettenhofer, P.; Weiss, R.; Dubourg, V.; et al. Scikit-learn: Machine Learning in Python. *J. Mach. Learn. Res.* **2011**, *12* (null), 2825–2830.

(9) Lange, A.; Herbert, J.; Albrecht, B.; You, Z.-Q. Intrinsically smooth discretisation of Connolly's solvent-excluded molecular surface. *Molecular Physics* **2019**, *118*, 1-18. DOI: 10.1080/00268976.2019.1644384.

(10) Wang, P.; Dhananjayan, N.; Hagras, M. A.; Stuchebrukhov, A. A. Respiratory complex I: Bottleneck at the entrance of quinone site requires conformational change for its opening. *Biochim Biophys Acta Bioenerg* **2021**, *1862* (1), 148326. DOI: 10.1016/j.bbabio.2020.148326 From NLM Medline.

(11) Dhananjayan, N.; Wang, P.; Leontyev, I.; Stuchebrukhov, A. A. Quinone binding in respiratory complex I: Going through the eye of a needle. The squeeze-in mechanism of passing the narrow entrance of the quinone site. *Photochem Photobiol Sci* **2022**, *21* (1), 1-12. DOI: 10.1007/s43630-021-00113-y From NLM Medline.

(12) Ponder, J. W.; Case, D. A. Force fields for protein simulations. *Adv Protein Chem* **2003**, *66*, 27-85. DOI: 10.1016/s0065-3233(03)66002-x From NLM Medline.

(13) Jorgensen, W. L.; Maxwell, D. S.; Tirado-Rives, J. Development and Testing of the OPLS All-Atom Force Field on Conformational Energetics and Properties of Organic Liquids. *Journal of the American Chemical Society* **1996**, *118* (45), 11225-11236. DOI: 10.1021/ja9621760.
